# Pilot study assessing the structural changes in posttrabecular aqueous humor outflow pathway after trabecular meshwork surgery using swept-source optical coherence tomography

**DOI:** 10.1371/journal.pone.0199739

**Published:** 2018-06-28

**Authors:** Munemitsu Yoshikawa, Tadamichi Akagi, Akihito Uji, Hideo Nakanishi, Takanori Kameda, Kenji Suda, Hanako Ohashi Ikeda, Akitaka Tsujikawa

**Affiliations:** Department of Ophthalmology and Visual Sciences, Kyoto University Graduate School of Medicine, Kyoto, Japan; Bascom Palmer Eye Institute, UNITED STATES

## Abstract

This study evaluated the morphological change in aqueous humor outflow (AHO) pathways using swept-source optical coherence tomography (SS-OCT) volumetric scans in glaucoma patients before and after glaucoma surgery. In this prospective observational case series, 15 eyes (13 patients) with glaucoma were treated with 120-degree Trabectome or 360-degree suture trabeculotomy and followed up for 3 months. B-scan images of the posttrabecular AHO pathway were reconstructed and the pathway areas were evaluated, before and after surgery. Changes in posttrabecular AHO pathway were qualitatively classified as “increased”, “non-significant change”, and “decreased” on reconstructed B-scan images. Quantitative measurements of the posttrabecular AHO pathway areas were performed pre- and postoperatively. Factors associated with both qualitative and quantitative changes in AHO pathway were investigated. From 30 regions (15 nasal and 15 temporal regions) in the 15 eyes, AHO pathways were analyzable in 20 regions pre- and postoperatively. Qualitative assessments of the pathway changes were “increased” in 8 regions, “non-significant change” in 9 regions, and “decreased” in 3 regions. Quantitative assessments of the average pathway area did not change significantly (from 3155±1633 pixels preoperatively to 3212±1684 pixels postoperatively, *P* = 0.50). All parameters relating to intraocular pressure changes or the surgical location were not associated with postoperative AHO pathway change. The intrascleral AHO pathway could be well visualized in glaucoma patients pre- and postoperatively using swept-source optical coherence tomography. However, structural changes in the AHO pathway assessed by SS-OCT were not significant after trabecular-targeted glaucoma surgery. Functional assessments of AHO are needed in future studies.

## Introduction

Glaucoma is a leading cause of preventable blindness worldwide, and intraocular pressure (IOP) control is an important factor for preventing glaucoma progression [1–3]. Trabecular-targeted minimally invasive glaucoma surgeries (MIGS) such as trabecular ablation using Trabectome (TAT) and 360-degree suture trabeculotomy (S-LOT) have gained popularity as IOP lowering treatments [4–11]. These surgeries are aimed at relieving the resistance of aqueous humor outflow (AHO) by means of removal or incision of the trabecular meshwork and inner layer of the Schlemm’s canal. Although MIGS have an advantage with regards to safety and rapidity, their variability and inconsistency in being able to lower IOP is a problem [12,13]. A good way to predict the IOP lowering effects of MIGS has been expected.

Several studies have tried to visualize the routes of AHO in the eyes of living human subjects. They include intraoperative observation using an operation microscope [14], intraoperative aqueous angiography using indocyanine green [15], or anterior-segment (AS-) optical coherence tomography (OCT) [16–19]. However, it has been difficult to visualize the total route of AHO noninvasively and it is not known whether the structures of the posttrabecular AHO pathway change after trabecular meshwork surgery. Recently, Uji et al. [20] reported that swept-source (SS-) OCT image processing using the motion contrast enhancement technique can visualize the three-dimensional whole AHO pathway from the Schlemm’s canal through the collector channel to the episcleral venous plexus. This method is expected to make it possible to compare the structural changes in the same AHO pathway of the same patients at different times. Structural assessments of the AHO before and after trabecular-targeted MIGS might provide important information for understanding the mechanism of their IOP lowering effects and possibly predict their surgical effects.

In this study, we prospectively investigated the morphological change in AHO pathways using SS-OCT volumetric scans in glaucoma patients before and after trabecular-targeted MIGS.

## Materials and methods

This prospective observational study was approved by the institutional review board and ethics committee of the Kyoto University Graduate School of Medicine and adhered to the tenets of the Declaration of Helsinki. According to the guidelines, written informed consent to participate in a prospective study was obtained from all the patients after the nature and possible consequences of the research had been explained to them.

### Patients

The candidates for this study were Japanese glaucoma patients who underwent TAT or S-LOT at Kyoto University Hospital between September 30, 2015 and October 7, 2016 and agreed to participate in this study. To be included, the candidates had to have open-angle glaucoma diagnosed by gonioscopy, have no history of intraocular surgery other than cataract surgery, and no other ocular disease that could affect the visual field. Patients were examined preoperatively and at 3 months postoperatively by SS-OCT (DRI OCT-1; Topcon, Tokyo, Japan). The exclusion criteria were as follows: presence of peripheral anterior synechiae, angle recession caused by trauma, neovascularization of the iris, and poor-quality SS-OCT images. Both eyes of the patients were included if eligible, using generalized estimating equation (GEE) models.

For the surgical procedures, TAT was performed and involved ablation of the regional 120 degrees of the trabecular meshwork, and S-LOT was performed with a 360 degree incision into the trabecular meshwork. All TAT and S-LOT surgeries were performed with a temporal clear corneal incision without making any incision into the sclera. Patients who underwent TAT or S-LOT combined with phacoemulsification cataract surgery with intraocular lens implantation were also included in this study.

### Ophthalmologic examinations

All patients underwent a comprehensive ophthalmologic examinations, including measurement of best-corrected visual acuity with a 5-m Landolt chart, slit-lamp biomicroscopy, gonioscopy, Goldman applanation tonometry, dilated stereoscopic examination of the optic nerve head and fundus, stereo disc photography (3-Dx simultaneous stereo disc camera; Nidek, Gamagori, Japan), axial length (AL) measurements by partial coherence interferometry (IOLMaster; Carl Zeiss Meditec, Dublin, CA, USA), standard achromatic automated perimetry (SAP) using the 24–2 Swedish Interactive Threshold Algorithm standard program (Humphrey Visual Field Analyzer; Carl Zeiss Meditec), and AHO imaging using DRI OCT-1.

### Visualization and measurement of the aqueous humor outflow pathway using the 1050-nm swept-source optical coherence tomography system

Details of AHO imaging have been described previously [20]. Briefly, SS-OCT with an axial scan rate of 100,000 Hz was used, and a three-dimensional volume scan mode containing 512 × 256 axial scans in a scan length of 3 × 3 mm was used for both the nasal and temporal sides of the corneal limbus for each subject. After flattening the images at the level of the conjunctival epithelium, en face images were generated using built-in software (Enview; Topcon). Subsequently, 202 frames spanning the episclera to the interface between the sclera and ciliary body were extracted, and after application of a Gaussian blur filter, 201 division images were calculated by dividing the pixels between sequential frames as [*D*_*j*_*(x*, *y) = I*_*j*_*(x*, *y)/I*_*j*_ *+*_1_*(x*, *y)*], where *I*_*j*_*(x*, *y)* represents the intensity of frame *j*. Next, the division images were divided into three equal stacks of frames, and for each stack, the variance of the pixels among all of the division images at each x-y position was calculated to visualize the contrast-enhanced vessel images. As a consequence, three vascular images of different depths were obtained, and the images were merged in different colors in order to visualize the connection between the layers [20]. Digital image processing was performed using ImageJ software (National Institutes of Health, Bethesda, MD; in the public domain: http://rsb.info.nih.gov/ij/index.html), and a series of ImageJ commands was performed automatically by means of a macro [20].

After three vascular images (vasculature map) were constructed using the above-mentioned motion contrast enhancement technique, both the vasculature maps and original en face images were used to newly reconstruct the B-scan images of the total AHO pathway. That is, a segmented line selection was made on the vascular image and copied to the en face images, followed by re-slicing the sequential three-dimensional images to obtain the new B-scan image. The Schlemm’s canal was identified as the dark, hyporeflective luminal structure that ran parallel to the limbus, and continuous cross-section of the vessels from Schlemm’s canal to the episcleral venous plexus was identified as the trajectory of dark, moving objects, using the en face images [20].

### Qualitative assessment

Structural changes in the AHO pathway were qualitatively evaluated before and after surgery by referring to the pre- and postoperative reconstructed B-scan images. Two authors (MY and TA, ophthalmologists with 7 and 20 years of experience, respectively) determined which image showed the larger AHO pathway while being blinded to the clinical information of the images. Then, the structural changes were categorized into 3 groups, “increased”, “non-significant change”, and “decreased”. Disagreements between these two authors were resolved by a third adjudicator (HN). The intraclass correlation coefficients (ICC [2, 1]) were calculated for the subjective categorizations of the postoperative AHO pathway. According to Fleiss, ICCs ≥ 0.75, between 0.40 and 0.75, and ≤ 0.4 were defined as excellent, moderate, and poor, respectively [21].

### Quantitative assessment

**Fig 1:**
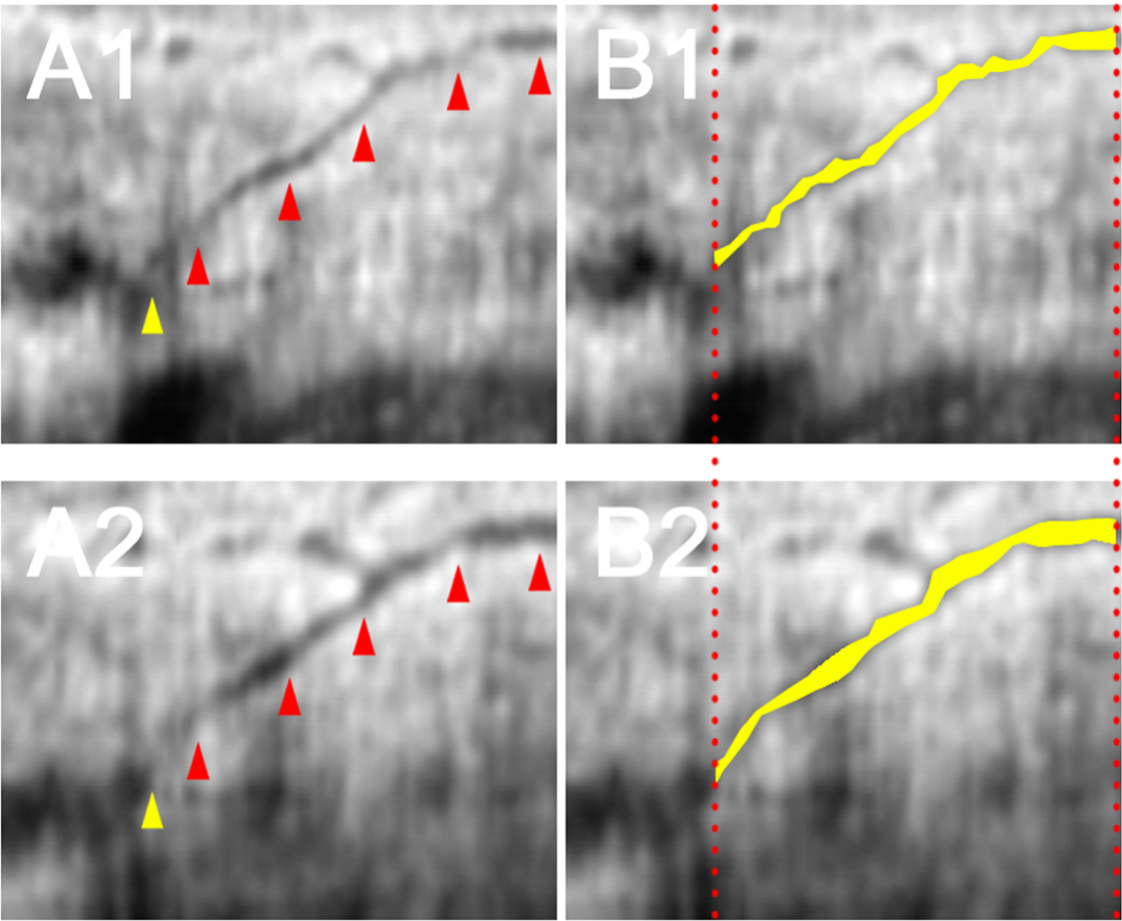
Illustration of objective measurement of the posttrabecular aqueous outflow pathway. Preoperative and postoperative reconstructed B-scan images of the same posttrabecular aqueous outflow pathway are shown in A1 and A2, respectively. The outflow pathways (red arrowhead) were measured from the Schlemm’s canal (yellow arrowhead) to the episcleral veins. (B1, B2) The measured area (yellow area) increased postoperatively from 5338 to 5714 pixels.

The area of the AHO pathway was measured on the reconstructed B-scan images from the entrance of the collector channel to the episcleral vein both pre- and postoperatively in the same regions of a same AHO pathway (Fig 1). The axial resolution could be defined as 2.6 μm/pixel, whereas the lateral resolution of the image was unknown because of the inaccuracy of the scan length. The measurements were calculated in pixel area.

### Statistical analyses

Statistical analyses were performed using R software (http://www.r-project.org/). A paired *t*-test was used to compare the pre- and postoperative values. Qualitative and quantitative changes in the AHO pathway were separately analyzed to account for the influence with several factors including age, sex, baseline IOP, percentage of IOP reduction, surgical procedure, surgically removed region, glaucoma diagnosis, cataract surgery, spherical equivalent refraction, AL, corneal curvature, central corneal thickness, visual field defect, and baseline AHO pathway area. For both univariate and multivariate analyses, generalized estimating equation (GEE) models were applied to incorporate the repeated measurements of paired-eye data [22,23]. Factors in the univariate analysis with a *P* < 0.20 were included in the multivariate analyses. For both quantitative and qualitative analyses, the level of significance was set at *P* < 0.05.

## Results

Initially, 15 eyes of 13 patients were included in this study. The posttrabecular AHO pathway was successfully identified in 25 out of the 30 regions of the 15 eyes (83%) preoperatively. Among the successfully identified AHO pathways preoperatively, the same postoperative AHO pathway was identified with comparable B-scans in 20 (80%) of these 25 regions. Two regions were excluded because of insufficient quality of postoperative SS-OCT images, and three regions were excluded due to the inability to identify the same AHO pathway in the preoperative and postoperative SS-OCT images. Demographics of the analyzed and excluded subjects are shown in Table 1.

**Table 1:** Patient demographics.

	Analyzed (n = 11)	Excluded (n = 2)
Age (years)	59.1 ± 18.1 (32 ― 78)	72.5 ± 0.7 (72 ― 73)
Sex (male/female)	2/9	2/0
Right eye/Left eye	5/8	1/1
Nasal side/Temporal side	11/9	1/2
Surgical procedure (TAT/S-LOT)*	9/4	1/1
Condition of trabecular meshwork (ablated or incised/intact)	13/7	2/1
Diagnosis (POAG/other)	8/3	1/1
Cataract surgery (YES/NO)	10/3	2/0
SER (diopters)	-4.48 ± 5.49 (-14.25 ― 3.25)	-2.19 ± 2.03 (-3.63 to -0.75)
Corneal curvature (mm)	7.52 ± 0.18 (7.23 ― 7.79)	7.63 ± 0.13 (7.54 ― 7.72)
Axial length (mm)	24.94 ± 1.76 (22.19 ― 27.19)	24.08 ± 0.30 (23.87 ― 24.29)
Corneal thickness (μm)	520.3 ± 23.5 (476 ― 547)	532.0 ± 21.2 (517 ― 547)
IOP preoperative (mmHg)	24.4 ± 9.8 (15 ― 48)	14.5 ± 0.7 (14 ― 15)
IOP postoperative (mmHg)	15.0 ± 3.2 (10 ― 20)	15.0 ± 2.8 (13 ― 17)
HFA MD (dB)	-11.15 ± 4.23 (-17.25 to -5.08)	-12.03 ± 1.27 (-12.92 to -11.13)

Abbreviations: HFA, Humphrey Field Analyzer; IOP, intraocular pressure; MD, mean deviation; POAG, primary open angle glaucoma; SER, spherical equivalent refraction.

* TAT, Trabectome surgery; S-LOT, 360-degree suture trabeculotomy.

**Fig 2:**
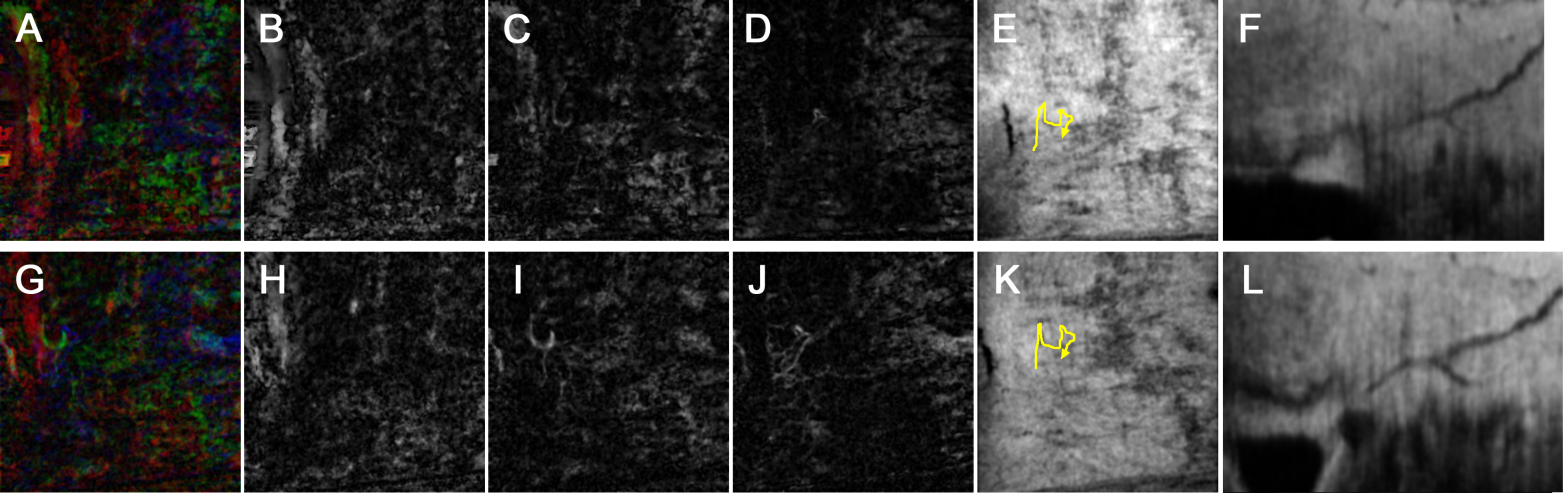
Representative images of the aqueous humor outflow pathway before and after surgery. The nasal limbus of a 77-year-old woman who received Trabectome surgery combined with cataract surgery in her right eye. Preoperative images (A-F) and postoperative images (G-L) are shown contrastively. (A, G) Merged images of the vasculature maps from three scleral layers in different colors (superficial layer (B, H), red; intermediate layer (C, I), green; deep layer (D, J), blue) for better visualization of the connection between the layers. (E, K) The identified aqueous outflow delineated on the en face image (yellow line). (F, L) Reconstructed B-scan obtained by re-slicing the volume scan data along the identified aqueous outflow pathway in the x-y plane (yellow line in E, K). Posttrabecular aqueous outflow pathways appeared as a dark line against the high-intensity scleral stroma. In this case, the pathway did not appear to change postoperatively.

**Fig 3:**
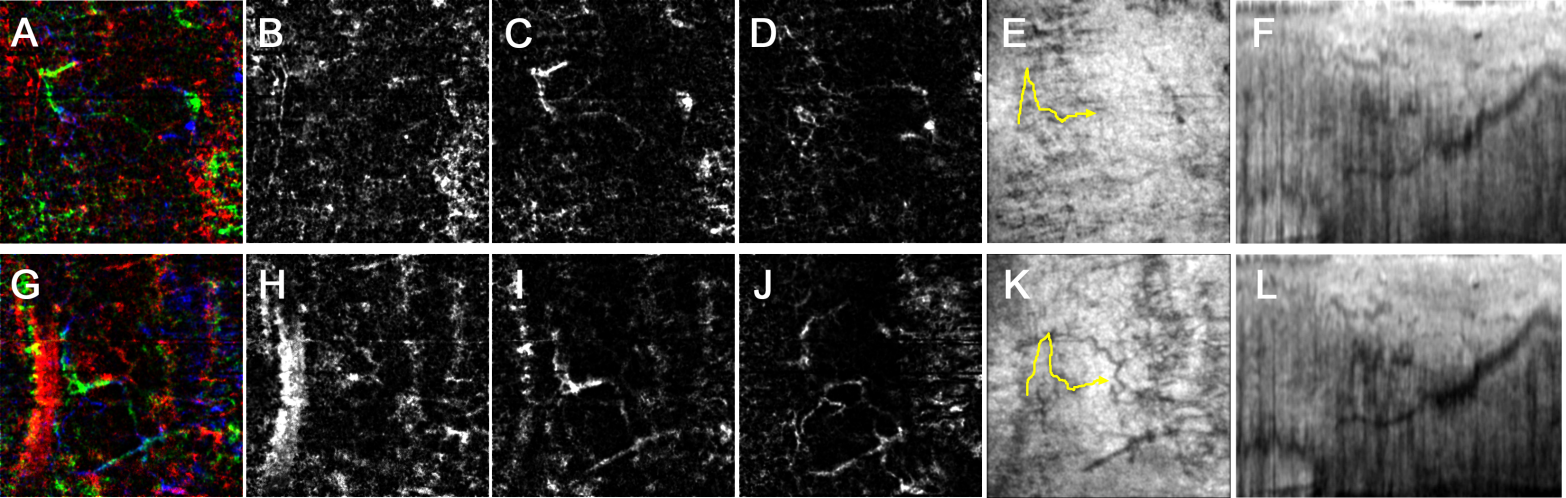
Representative images of the aqueous humor outflow pathway before and after surgery. The temporal limbus of a 32-year-old woman who received Trabectome surgery in her left eye. Preoperative images (A-F) and postoperative images (G-L) are shown contrastively. (A, G) Merged images of the vasculature maps from three scleral layers in different colors (superficial layer (B, H), red; intermediate layer (C, I), green; deep layer (D, J), blue). (E, K) The identified aqueous outflow delineated on the en face image (yellow line). (F, L) Reconstructed B-scan obtained by re-slicing the volume scan data along the identified aqueous outflow pathway in the x-y plane (yellow line in E, K). (F, L) Posttrabecular aqueous outflow pathway width seems to increase postoperatively.

In the 13 eyes included in the analysis, the IOP decreased from 26.1 mmHg to 14.7 mmHg after trabecular-targeted MIGS. In our qualitative assessments, structural changes in the AHO pathway were categorized as “increased” in 8 regions, “non-significantly changed” in 9 regions, and “decreased” in 3 regions. In our quantitative assessments, the average AHO pathway area was 3155±1633 pixels preoperatively and 3212±1684 pixels postoperatively (*P* = 0.50; paired *t*-test). Vasculature maps and reconstructed B-scan images of the pre- and postoperative AHO pathways are shown in Figs 2 and 3.

The postoperative structural changes in AHO were compared between at the trabecular-ablated/incised area and the trabecular-intact area (Table 2). Both qualitative and quantitative assessments of the AHO pathway width showed no significant differences between the areas. The interobserver reproducibility of the qualitative evaluation was excellent (ICC [2, 1] = 0.803) between the two examiners.

**Table 2:** Surgical location and change in the posttrabecular pathway.

	Removed	Unremoved	P*
Quantitative			
AHO pathway area—pre (pixel)	3277±2053	2928±605	0.67
AHO pathway area—post (pixel)	3308±2125	3033±609	0.74
*P*†	0.80	0.16	
AHO pathway area change (%)	0.6±7.8	3.9±5.7	0.34
Qualitative			
AHO pathway change (increased/non-significant/decreased)	5/5/3	3/4/0	0.43
IOP—pre (mmHg)	21.5±6.6	28.7±11.8	0.094
IOP—post (mmHg)	15.5±2.8	13.6±3.0	0.16
IOP change (%)	-21.5±25.1	-43.4±31.1	0.10

Abbreviations: AHO, aqueous humor outflow; IOP, intraocular pressure.

* unpaired *t*-test comparing Removed and Unremoved.

† paired *t*-test comparing preoperative and postoperative AHO pathway areas.

**Fig 4:**
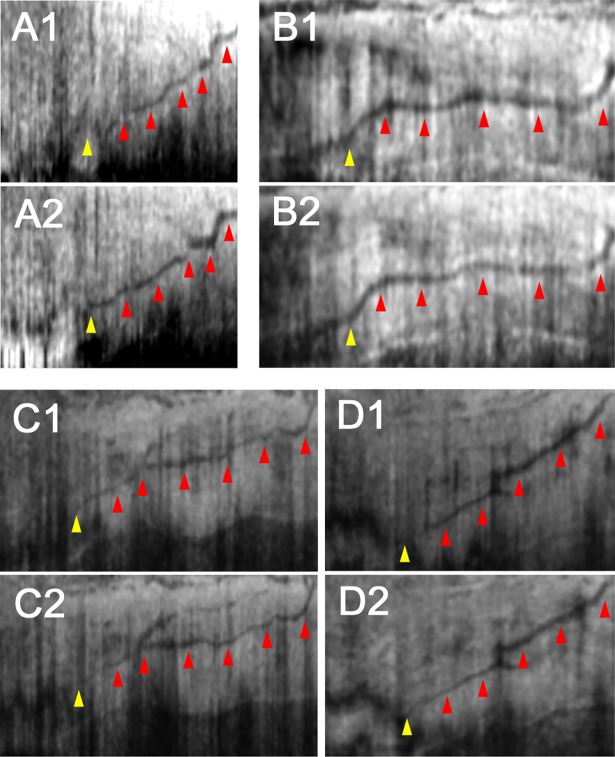
Representative images of identified posttrabecular aqueous humor outflow pathways before and after surgery. Preoperative (A1-D1) and postoperative (A2-D2) AHO pathways (*red arrowhead*) were shown to be connected to the Schlemm’s canal (*yellow arrowhead*). (A1, A2) The nasal limbus of a 63-year-old woman who received Trabectome surgery combined with cataract surgery in her left eye. (B1, B2) The temporal limbus of a 62-year-old man who received 360-degree suture trabeculotomy ab interno combined with cataract surgery in his left eye. (C1, C2) The temporal limbus of a 57-year-old woman who received Trabectome surgery combined with cataract surgery in her left eye. (D1, D2) The temporal limbus of a 77-year-old woman who received Trabectome surgery combined with cataract surgery in her right eye. Postoperative outflow pathways were qualitatively categorized as increased (A), decreased (B), and non-significant change (C, D). The quantitative measurements of the AHO pathway area were 1796 (A1), 1848 (A2), 7595 (B1), 6643 (B2), 3316 (C1), 3069 (C2), 2522 (D1), and 2758 (D2) pixels, respectively.

Factors that could be associated with AHO pathway change are shown in Table 3 and Table 4, respectively. In the qualitative assessments, no parameter was significantly associated with changes in the postoperative AHO pathway (Table 3). In the quantitative assessments, univariate analyses showed that gender (*P* = 0.012) and combined cataract surgery (*P* = 0.042) were significantly associated with changes in postoperative AHO pathway. In the multivariate analysis, only female gender was significantly associated with a postoperative increase in AHO pathway (*P* = 0.026). No other parameters were significantly associated with changes in postoperative AHO pathway. The representative images showing AHO pathway changes are shown in Fig 4.

**Table 3:** Univariate and multivariate analysis of factors associated with qualitative changes in postoperative aqueous humor outflow pathway width.

	Univariate		Multivariate	
	β (95% CI)†	*P*†	β (95% CI)*	*P*†
Age, per 1-y older	0.01 (-0.01―0.02)	0.41		
Female gender (vs. male)	-0.45 (-1.85―0.95)	0.53		
Baseline IOP, per 1-mmHg higher	-0.00 (-0.04―0.03)	0.76		
% IOP change, per 1% higher	0.33 (-0.71―1.38)	0.53		
Suture trabeculotomy (vs. Trabectome)	0.48 (-0.32―1.28)	0.24		
Surgically removed location (vs. unremoved location)	0.20 (-0.18―0.57)	0.30		
POAG subtype (vs. secondary glaucoma subtype)	0.11 (-0.39―0.62)	0.66		
Combined with cataract surgery (vs. alone)	0.35 (-0.26―0.95)	0.26		
Axial length, per 1-mm longer	-0.04 (-0.21―0.13)	0.63		
SER, per 1-diopter more hyperopic	0.02 (-0.04―0.08)	0.47		
Corneal curvature radius, per 1-mm higher	-1.02 (-2.87―0.83)	0.28		
Central corneal thickness, per 1-μm thicker	0.00 (-0.02―0.01)	0.87		
HFA MD, per 1-dB better	0.06 (-0.02―0.14)	0.17	0.06 (-0.02―0.14)	0.17

Abbreviations: IOP, intraocular pressure; POAG, primary open angle glaucoma; SER, spherical equivalent refraction; HFA, Humphrey Field Analyzer; MD, mean deviation.

* Effect sizes for the dependent variable of a qualitative change.

† Generalized estimating equation analysis.

**Table 4:** Univariate and multivariate analysis of factors associated with quantitative changes in postoperative aqueous humor outflow pathway width.

	Univariate		Multivariate	
	β* (95% CI)	*P*†	β* (95% CI)	*P*†
Age, per 1-y older	-0.00 (-0.00―0.00)	0.44		
Female gender (vs. male)	0.08 (0.02―0.14)	0.012	0.06 (0.01―0.11)	0.026
Baseline IOP, per 1-mmHg higher	0.00 (-0.03―0.03)	0.96		
% IOP change, per 1% higher	-0.06 (-0.14―0.02)	0.17	0.03 (-0.17―0.22)	0.80
Suture trabeculotomy (vs. Trabectome)	-0.03 (-0.10―0.04)	0.43		
Surgically removed location (vs. unremoved location)	-0.03 (-0.09―0.03)	0.27		
POAG subtype (vs. secondary glaucoma subtype)	0.00 (-0.06―0.06)	0.97		
Combined with cataract surgery (vs. alone)	-0.04 (-0.07 to -0.00)	0.042	-0.04 (-0.15―0.07)	0.48
Axial length, per 1-mm longer	0.00 (-0.01―0.01)	0.97		
SER, per 1-diopter more hyperopic	-0.00 (-0.01―0.00)	0.74		
Corneal curvature radius, per 1-mm higher	-0.04 (-0.19―0.11)	0.62		
Central corneal thickness, per 1-μm thicker	-0.00 (-0.00―0.00)	0.34		
HFA MD, per 1-dB better	-0.00 (-0.01―0.00)	0.14	-0.00 (-0.01―0.00)	0.21
Baseline AHO area, per 1-pixel larger	0.00 (-0.00―0.00)	0.95		

Abbreviations: IOP, intraocular pressure; POAG, primary open angle glaucoma; SER, spherical equivalent refraction; HFA, Humphrey Field Analyzer; MD, mean deviation; AHO, aqueous humor outflow.

*Effect sizes for the dependent variable of a quantitative change.

† Generalized estimating equation analysis.

## Discussion

In the current study, we investigated structural changes in the posttrabecular AHO pathway following IOP reduction after trabecular-targeted MIGS. The posttrabecular AHO pathways in the same patients could be well visualized in B-scan images reconstructed from SS-OCT volume scans before and after surgery. However, in both qualitative and quantitative assessments, no significant differences in the AHO pathway areas could be detected from before and after the MIGS.

The main resistance of AHO is thought to be located in the trabecular meshwork and the Schlemm’s canal and this resistance is increased in glaucomatous eyes [24–27]. Trabecular-targeted MIGS can remove the resistance at the level of the trabecular meshwork; however, trabecular-targeted MIGS could not reduce the IOP to the level of episcleral venous pressure (generally 7―8 mmHg) [4,28–30]. This discrepancy is thought to be derived from the existence of posttrabecular outflow resistance, which was accounted for microstructures such as collector channels [24,31,32]. Furthermore, IOP lowering by trabecular-targeted MIGS is well known to be variable and inconsistent. Imaging of the posttrabecular AHO pathway might provide us with a better understanding of glaucoma surgery.

Fellman et al. [14] reported that a washout wave in the episcleral vein can be intraoperatively evaluated and predict obstruction or sufficient flow through the collector channels. Huang et al. [15] demonstrated intraoperative aqueous angiography using indocyanine green (ICG) can visualize routes of AHO. They showed that trabecular-targeted MIGS using trabecular bypass stents could increase aqueous angiography in an enucleated human eye [26]. These two studies suggest that the assessment of aqueous flow in the posttrabecular AHO pathway might be useful for evaluating the effectiveness of trabecular-targeted MIGS. However, these methods have some disadvantages including that they need to be performed in the operating room and difficulty with quantitative assessments. Therefore, non-invasive, convenient, quantitative assessments of the posttrabecular AHO pathway are required.

It also has been reported that the microstructure of the Schlemm’s canal and collector channel could be visualized using AS-OCT [17–20,33]. On AS-OCT images, the AHO pathway can be detected as the intrascleral lumen, which corresponded with angiographically positive regions [34]. However, it should be noted that the intrascleral lumen visualized by AS-OCT does not always guarantee the existence of a functional AHO and it is unknown whether the increase of AHO induces enlargement of the intrascleral lumen. Our non-invasive method enabled repeated examinations and comparison of the same AHO pathway in the same patient. However, no significant increases in the AHO pathway were detected after successful trabecular-targeted MIGS. Our results suggest that postoperative changes in the AHO pathway might be too small for our method to detect, or the width of the intrascleral lumen might not change even when the AHO increases. It was previously suggested that increased AHO can lead to the expansion of the Schlemm’s canal [34]. The inner wall of the Schlemm’s canal is likely to be flexible as it is in contact with the aqueous humor of the anterior chamber. On the other hand, the intrascleral lumen might be less flexible than the Schlemm’s canal because as it is surrounded by scleral tissue, which is relatively stiff. Further studies are needed to clarify the relationship between the function and structures in AHO pathways.

The current study has several limitations. First, our method enabled comparison of the same AHO pathway thoroughly from the Schlemm’s canal through the collector channel to the episcleral venous plexus at different times. However, it should be noted that only one AHO pathway, and not all AHO pathways, was selected in the current study. This is important because some of the AHO structures are thought to be dynamic in nature [34]. Further studies are needed to evaluate postoperative changes in all AHO pathways. Second, we were only able to identify the same AHO pathway postoperatively in only 80% of the regions where we could identify posttrabecular AHO pathway preoperatively. If the AHO pathway had significantly decreased in a region, it might have been excluded from further analyses, which may have led to the overestimation of the increase in AHO pathway. However, even if this was the case, it should not affect our results. Third, although we showed the potential of AS-OCT to reproducibly visualize the AHO pathway, valid comparison between pre and postoperative images requires a higher level of imaging. Even when pre- and postoperative AHO images look very similar, close attention should be paid to the possibility that the differences in registration, focus and clarity between the paired images might at least partially affect the ability to identify significant changes.

In conclusion, the intrascleral AHO pathway was well visualized in glaucoma patients pre- and postoperatively using SS-OCT. There were no significant structural changes in the posttrabecular AHO pathway after trabecular-targeted MIGS. Further studies assessing functional AHO are needed to clarify the relationship between trabecular-targeted MIGS and the posttrabecular AHO pathway.

## Supporting information

S1 Dataset The raw data of all subjects. (XLSX)
